# Diffuse scattering and partial disorder in complex structures

**DOI:** 10.1107/S205225251402065X

**Published:** 2014-10-28

**Authors:** T. R. Welberry, D. J. Goossens

**Affiliations:** a Research School of Chemistry, Australian National University, Canberra, ACT 0200, Australia

**Keywords:** single-crystal diffuse scattering, disorder, synchrotron light sources

## Abstract

The study of single-crystal diffuse scattering (SCDS) goes back almost to the beginnings of X-ray crystallography. Because SCDS arises from two-body correlations, it contains information about local (short-range) ordering in the sample, information which is often crucial in the attempt to relate structure to function. This review discusses the state of the field, including detectors and data collection and the modelling of SCDS using Monte Carlo and *ab initio* techniques. High-quality, three-dimensional volumes of SCDS data can now be collected at synchrotron light sources, allowing ever more detailed and quantitative analyses to be undertaken, and opening the way to approaches such as three-dimensional pair distribution function studies (3D-PDF) and automated refinement of a disorder model, powerful techniques that require large volumes of low-noise data.

## Abstract 2

This review discusses the state of the field of single-crystal diffuse scattering (SCDS), including detectors, data collection and the modelling techniques. High quality, three-dimensional volumes of SCDS data can now be collected at synchrotron light sources, allowing increasingly detailed and quantitative analyses to be undertaken.

## 1. Introduction   

The strong sharp Bragg reflections that occur in diffraction patterns of all real crystals are used by conventional X-ray crystallography to deduce the average repetitive arrangements of atoms or molecules. Diffuse scattering, on the other hand, contains information about the deviations from the average (*i.e.* different types of disorder) and gives structural information on a scale that goes beyond that of the average unit cell and extends over a range of ∼ 1–1000 Å. In many important materials, it is this extended range of structural information that is crucial in determining the unique or novel properties of the material, rather than the average unit-cell structure.

By determining the local structural detail of such materials a much better understanding can be gained of why they have the properties they do and new materials may be identified or designed. Some examples of materials whose properties are intimately associated with the disordered nature of their structure are:

1. High-temperature ceramics such as cubic zirconias (Gallardo-López *et al.*, 2001 ▶; Frey *et al.*, 2005 ▶) and mullite (Lazic *et al.*, 2013 ▶). Cubic zirconia has a simple cubic average, but the arrays of oxygen atoms contain vacancies and the metal atoms relax around these (Welberry *et al.*, 1995 ▶). The vacancies provide the means for superionic conductivity and phase stability over a wide composition range.
2. Alloys including quasi-crystals. Consisting as they do of mixtures of different atomic species, alloys are intrinsically disordered (Matsubara & Cohen, 1985*a* ▶,*b* ▶; Frey & Steurer, 1985 ▶; Schaub *et al.*, 2011 ▶; Yamamoto, 2010 ▶; Abe *et al.*, 2003 ▶; Weidner *et al.*, 2004 ▶). Details of local atomic ordering influence such properties as hardness, tensile strength and electrical and thermal conductivity.
3. Catalytic materials including zeolites. Zeolites are large open framework structures containing channels and cavities. Not only do the shapes and sizes of the channels and cavities influence the material’s ability to discriminate between different adsorbing molecules, but it is clear that the flexibility of the cavities (causing local departures from regularity) is important too (Nenoff *et al.*, 1996 ▶; Abeykoon *et al.*, 2008 ▶; Campbell *et al.*, 2004 ▶; Yoshida *et al.*, 2013 ▶).
4. Liquid crystals. Liquid crystals represent a state of order intermediate between truly crystalline and truly liquid. Many different types exist involving varying degrees of short and long-range translational and orientational order (Brasselet & Galstian, 2000 ▶; Guégan *et al.*, 2006 ▶; Lefort *et al.*, 2008 ▶; Chahine *et al.*, 2010 ▶).
5. Organic guest/host systems (inclusion compounds). Here the host lattice provides cavities where introduced guest species can be confined in environments not normally experienced by the pure guest material. This allows the potential for control of physical properties and chemical reactivity (Welberry & Mayo, 1996 ▶; Weber *et al.*, 2001*a* ▶; Bürgi *et al.*, 2005 ▶; Harris, 2003 ▶; Mayo *et al.*, 1999 ▶). Examples include optical guest molecules, one-dimensional conducting materials, polymerization. Since the guest and host are not strongly bonded the relationship between guest and host is often disordered.
6. Macromolecular and protein structures. Structure and function are intimately tied together for these large molecules, but frequently it is not the average structure that is crucial but how different regions of the molecules move to provide access to active sites (Clarage & Phillips, 1997 ▶; Welberry *et al.*, 2011 ▶; Wall, 2009 ▶). For some protein structures as much as 50% of the total scattering is diffuse scattering.
7. Superconductors. Some theories on the origin of superconductivity in the high-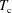 oxide superconductors attribute the effect to the glassy state of flux lines originating from the disorder in the oxygen/vacancy arrays (Matsuda *et al.*, 2000 ▶). The complex structures of the materials show many sources of diffuse scattering (Le Tacon *et al.*, 2014 ▶; Schrieffer, 2007 ▶).
8. Pharmaceuticals. There is much topical interest in polymorphism of pharmaceutical drugs (Chan & Goossens, 2012 ▶; Chan, Welberry *et al.*, 2009 ▶; Rodríguez-Spong *et al.*, 2004 ▶; Parmar *et al.*, 2007 ▶; Schmidt, 2005 ▶; Plano *et al.*, 2011 ▶) and other small molecules of biological relevance, including foodstuffs (Mathlouthi *et al.*, 2012 ▶). There is evidence that disorder is of importance in explaining the occurrence of polymorphism.
9. Relaxor ferroelectrics. Relaxors have attracted tremendous attention in recent years because of their extraordinary dielectric and piezoelectric properties (Park & Shrout, 1997 ▶; Welberry & Goossens, 2008 ▶; Goossens, 2013 ▶; Chernyshov *et al.*, 2011 ▶). It is generally accepted that their structure is comprised of nanometer-sized polar domains or nanoregions, PNRs (Burns & Dacol, 1983 ▶), and it is widely believed that these play an essential role in the relaxor properties (Cross, 1987 ▶), although there are some doubts (Hlinka, 2012 ▶; Paściak *et al.*, 2013 ▶; Paściak & Welberry, 2011 ▶). The discussion is vibrant.

## 2. Measurement of diffuse X-ray scattering   

Despite the fact that diffuse scattering was observed from the earliest years of X-ray diffraction, the development of methods to interpret and analyze it lagged well behind the development of conventional average structure determination using the Bragg peaks. The reasons for this are that diffuse scattering intensities are much weaker than Bragg peaks, making the experimental observation vastly more demanding and time consuming. However, the advent of intense synchrotron sources and various kinds of area detectors means that this aspect of the problem is largely solved, and it is now possible to obtain high-quality three-dimensional diffuse scattering data relatively routinely (Welberry, Goossens *et al.*, 2005 ▶; Chan, Welberry *et al.*, 2009 ▶). The second reason is that the sheer diversity of different types of disorder that occur in nature has made it difficult to formulate a solution strategy that will work for all problems.

### 2.1. Historical   

**Figure 1:**
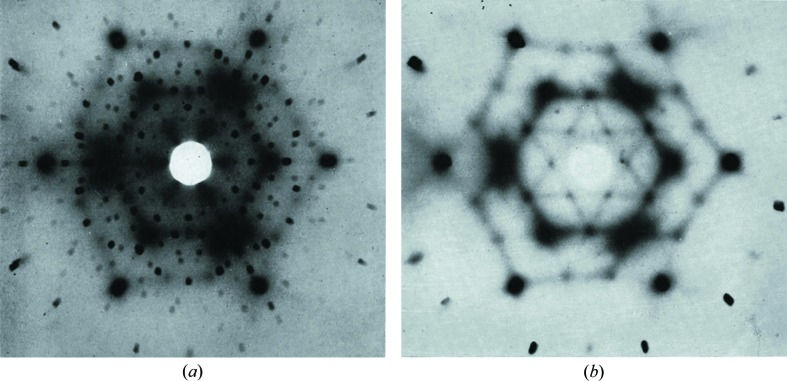
Laue photographs of benzil, C_14_H_10_O_2_, adapted from plate 8 of Lonsdale & Smith (1941 ▶). The incident beam is approximately along [0001]. (*a*) Using unfiltered Cu radiation. (*b*) Using monochromatic Cu *K*α radiation.

For the first 50 years of the development of crystallography use of photographic recording methods was the norm. This undoubtedly contributed to the fact that experimenters were well aware of diffuse scattering and there was much interest in it. As an example of the kind of detailed diffuse scattering that could be observed Fig. 1 ▶ shows data recorded by Lonsdale & Smith (1941 ▶). The first issue of *Acta Crystallographica* provides a snapshot of the range of crystallographic interests and activities in 1948 ▶ and studies of diffuse scattering feature quite prominently. Indeed the very first paper (Garrido, 1948 ▶) was a study of diffuse scattering of X-rays by crystals of NaClO_3_ and in the same volume there were numerous other papers concerned with diffuse scattering. Although the main focus of crystallography at this stage was still to solve the phase problem for crystal structure determination from the Bragg peaks, diffuse scattering was seen as valuable additional information that could be used to aid the solution.

The diffraction patterns shown in Fig. 1 ▶ are stationary crystal Laue photographs recorded by placing a film behind the crystal upon which a collimated beam of X-rays is incident. When only a single wavelength is present (Fig. 1 ▶ *b*) the image comprises a projection of the curved section of reciprocal space corresponding to the intersection of the film with the Ewald sphere.

With the advent of computers and automatic single-crystal diffractometers in the 1950s (Bond, 1955 ▶; Benedict, 1955 ▶) the measurement and analysis of Bragg intensities was revolutionized. While this enormously stimulated the development of crystal structure determination, it also led to a diminution of the study of diffuse scattering, since it was now possible for researchers to collect Bragg peak intensities without ever seeing the rest of the reciprocal space. However, with the development of Image Plates and other forms of one- and two-dimensional multi-detectors, together with the much enhanced intensity afforded by synchrotron beams, researchers have again become aware of the diffuse scattering in their samples and there is burgeoning interest in using it to explore structure on length scales that go beyond the average unit cell.

### 2.2. Current methods   

Basically the same method as that used by Lonsdale & Smith (1941 ▶) to record the data shown in Fig. 1 ▶(*b*) is still used today, though with much enhanced intensities afforded by synchrotron sources, better monochromatization, smaller angular spread of the collimated beam and greater sensitivity of modern area detectors. Instead of the 2 h exposure required for Fig. 1 ▶(*b*), exposures for a single frame are now typically only a few seconds. By taking a series of such exposures as the crystal is rotated a complete three-dimensional volume of reciprocal space can be recorded.

**Figure 2:**
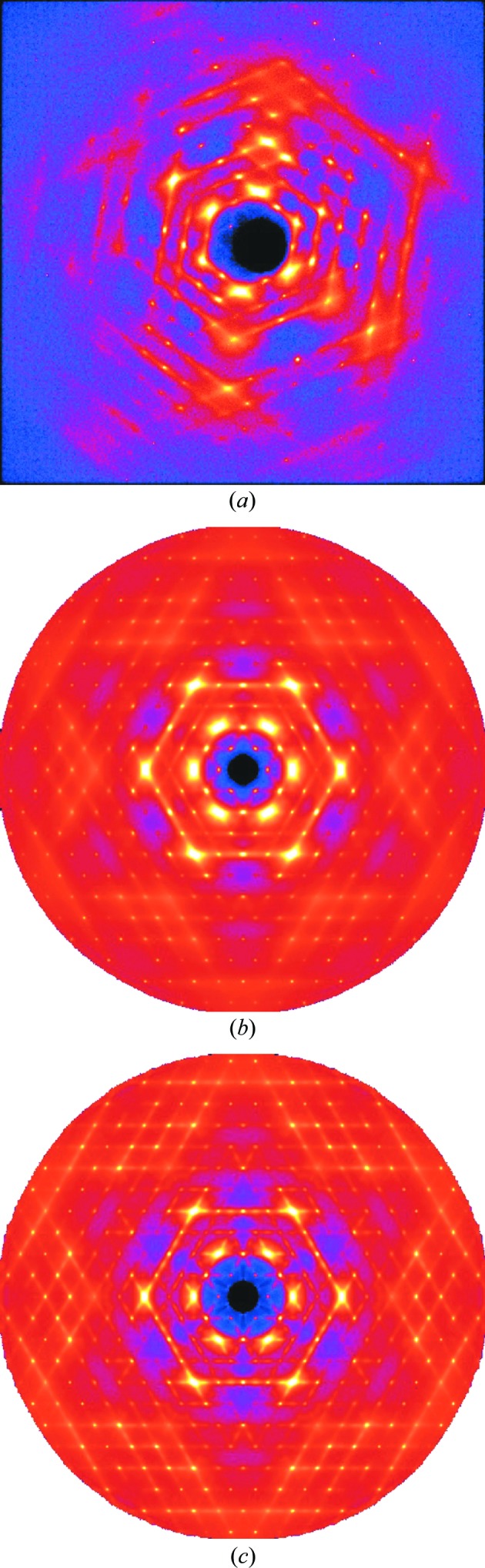
Diffraction patterns of benzil obtained from data recorded at the 11-ID-B beamline of the Advanced Photon Source (APS). (*a*) A single data frame recorded on a Perkin–Elmer amorphous silicon two-dimensional area detector using 58.26 keV X-rays (λ = 0.2127 Å). (*b*) The *hk*0 reciprocal section at 300 K, reconstructed from a data set comprising 740 such frames. (*c*) The same section recorded at 100 K. The maximum 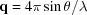 recorded was 8.52 Å^−1^. Note that the intensities in these images are displayed on a logarithmic scale.

Fig. 2 ▶ shows single-crystal diffuse scattering (SCDS) patterns of benzil recorded recently using a Perkin–Elmer amorphous silicon two-dimensional area detector at the 11-ID-B beamline of the Advanced Photon Source. A full three-dimensional volume of reciprocal space data was recorded comprising 740 individual frames in incremental steps of Δϕ = 0.25°, where ϕ is the crystal rotation angle. Fig. 2 ▶(*a*) shows a single frame of data recorded with an exposure time of 10 s, while Fig. 2 ▶(*b*) shows a reconstruction of the *hk*0 reciprocal section. The whole three-dimensional data collection took only slightly more than the 2 h used to record the single exposure shown in Fig. 1 ▶(*b*). Fig. 1 ▶(*c*) shows the same section of data recorded at 100 K.

Despite the ability to collect three-dimensional volumes of data these data have not generally been fully utilized in subsequent studies. Rather than making use of the whole three-dimensional data set most studies have tended to use a limited number of two-dimensional plane sections extracted from the three-dimensional data, even though the computer modelling used in the analyses might itself be fully three-dimensional. These two-dimensional sections have typically been extracted using purpose written software (see for example Estermann & Steurer, 1998 ▶; Maisel *et al.*, 2013 ▶) and may be zero-level sections normal to particular prominent zone axes, comparable upper-level sections normal to the same axes or non-integral sections that contain no Bragg peaks. One of the reasons for this use of two-dimensional plane sections is that they are more easily interpreted because they relate directly to specific simple projections of the structure. This considerably aids the initial modelling process since it is easier to relate particular diffraction effects to particular structural features.

Once a model has been decided, however, the model parameters could equally well be determined by fitting to the curved reciprocal lattice sections that correspond to the individual recorded frames. This strategy has been used for example in a study of phonon diffuse scattering in Si (Wu *et al.*, 1999 ▶; Xu & Chiang, 2005 ▶). If high-energy X-rays are used and the Ewald sphere has a very large radius a single exposure with the incident beam along a prominent zone axis gives a resultant diffraction pattern very similar to the corresponding zero-level plane section. This has been particularly useful in situations where experimental constraints preclude a complete data collection, for example experiments in an electric field (Xu *et al.*, 2006 ▶; Daniels *et al.*, 2006 ▶) or at high pressures (Kreisel *et al.*, 2003 ▶).

### 2.3. Problems in measuring diffuse X-ray scattering   

The rapid acquisition of high-quality three-dimensional diffuse scattering data that has resulted from the advent of electronic two-dimensional detectors has not been achieved without the need to overcome a number of technical issues. A variety of different types of detectors have been used ranging from position-sensitive wire proportional counters, image-plate detector/scanners, CCDs, amorphous silicon (Nathan *et al.*, 2000 ▶) or CMOS Flat Panel Detectors and single-photon-counting X-ray pixel detectors (Broennimann *et al.*, 2006 ▶; Kraft *et al.*, 2009 ▶). Despite the fact that the different types of detector use different physical effects (Arndt, 1986 ▶) to detect the incident X-rays, they all have limitations on the count rates that can be handled. Most detectors available to date provide 16-bit resolution which is insufficient to cover the dynamic range required to measure both diffuse scattering and Bragg peaks. If exposures are made to give acceptable signal-to-noise for the diffuse scattering many Bragg peaks will exceed the maximum count rate. The overflows that result cause various kinds of artefacts (*e.g.* blooming, ghosting *etc.*) that must be eliminated from the data by discarding affected regions (Welberry, Goossens *et al.*, 2005 ▶). In some cases the affected regions may be replaced by data recorded in a symmetrically related region of the pattern or by data recorded in a separate experiment.

Recent single-photon-counting X-ray pixel detectors such as the PILATUS (Broennimann *et al.*, 2006 ▶; Kraft *et al.*, 2009 ▶) provide 20-bit resolution and are therefore less prone to (though not completely immune from) producing artefacts but so far these are not available for high-energy X-rays. The dynamic range of the linear (one-dimensional) Mythen detector (Bergamaschi *et al.*, 2010 ▶) is even better, but its one-dimensional nature precludes its use for rapid acquisition of full three-dimensional diffuse scattering data.

## 3. Interpretation and analysis of diffuse scattering   

Although interpretation and analysis of diffuse scattering from single crystals remains a challenging problem, many advances have been made. Diffuse scattering arises in many different forms in a wide and diverse range of different materials and may be caused by many different physical or chemical effects. Consequently there has not been, until relatively recently, a single method that can be applied to interpret and analyse it. However, the advent of computer simulations in the last 25 years or so, together with the enormous increase in available computer power, has for the first time provided such a single method that can now be used to tackle virtually any disorder problem irrespective of the complexity of the structure, the form of the diffuse scattering or its cause. Such use of computer simulations of a model structure has become a powerful and well accepted technique for this purpose (Welberry & Butler, 1994 ▶; Welberry, 2004 ▶; Weber & Bürgi, 2002 ▶; Weber *et al.*, 2001*b* ▶). The method consists of comparing diffraction patterns calculated from a computer model of the disordered structure with measured X-ray or neutron diffuse intensities. The advantage of the method is that it can be applied generally to all systems, regardless of their complexity or the magnitude of the atomic displacements that might be present. The only limitation is the extent to which the model system can be made to provide a realistic representation of the real system. At one extreme, a very simplified model may be useful in providing a qualitative demonstration of particular effects (Welberry, 2001 ▶), while at the other extreme, a quantitative and detailed description of a disordered structure can be obtained (Welberry *et al.*, 2001 ▶).

### 3.1. Monte Carlo simulation of molecular crystals   

Monte Carlo simulation of the diffuse scattering in molecular crystals is an area where the most quantitative modelling has been achieved. The atomic positions in the simulation, based on coordinates from the average crystal structure, are specified in terms of bond lengths, bond angles and dihedral angles using a z-matrix description. The molecules may then be treated as a number of rigid fragments with the only flexibility allowed arising from variation of the dihedral angles associated with single chemical bonds.

The intermolecular interactions are modeled using harmonic (Hooke’s law) springs, with force constants 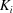, placed along a carefully selected subset of the large number of atom–atom vectors that typically make up a given intermolecular interaction
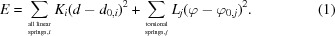
Here 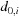 is the equilibrium length of the *i*th spring and *d* its instantaneous length. Similarly, 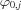 is the equilibrium value of the *j*th torsional angle and ϕ its instantaneous value. 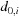 is assumed to be equal to the interatomic distance and 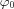 to the value of the particular torsion angle, observed in the average crystal structure.

**Figure 3:**
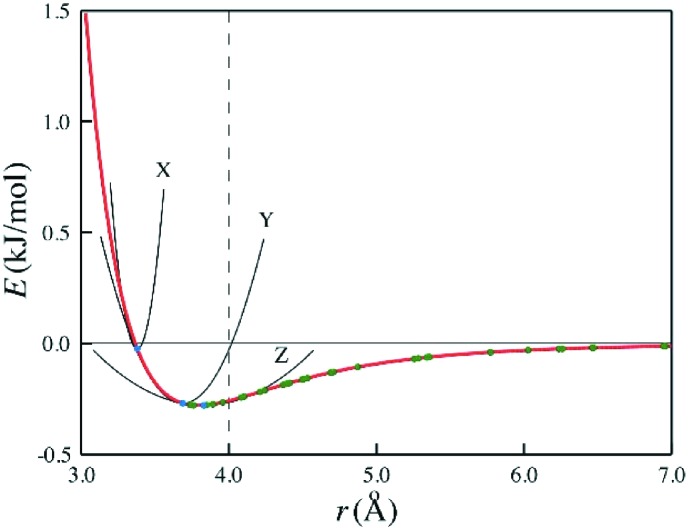
A Buckingham potential curve for a C—C non-bonded interaction. The superimposed coloured dots correspond to the lengths of individual C—C bonds comprising a particular intermolecular interaction. Reproduced from Chan, Welberry, Goossens *et al.* (2010 ▶) ▶ with permission.

In principle, the values of the spring constants 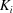 are parameters to be determined by fitting to the observed data. Recently a method has been developed whereby a simple functional form for their values, involving a few adjustable parameters, is used (Chan, Welberry, Goossens *et al.*, 2010 ▶). Fig. 3 ▶ shows a plot of a typical interatomic pair potential upon which has been superimposed the actual atom–atom distances that occur for all of the pairs making up a particular intermolecular interaction. These vary from very short contacts in which the individual atoms are pressed hard up against the repulsive exponential barrier to much longer ones out in the region where the softly attractive part of the potential dominates. For the shortest contact distance a participating atom will immediately encounter the steep repulsive wall if it is displaced towards its neighbour, and a large value for 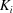 is expected. For longer vectors successively weaker force constants should be expected. These considerations have led to the formulation of a simple algorithm [equation (2)] by which a large number of potentially independent 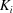 may be defined in terms of a few refinable parameters
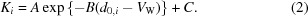
Here 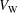 is the sum of the van der Waals radii (Bondi, 1964 ▶) for the two linked atoms, 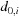 is the average length of a particular contact and *A*, *B* and *C* are constants to be determined by the fitting. The exponential form was adopted because the slope of the Buckingham potential increases exponentially as the repulsive wall is approached. The term 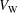 was included to take account of the differences in the sum of the van der Waals radii for different types of atom pairs. Formulated in this way the same values of *A*, *B* and *C* may be used for different types of atom–atom contact, but this constraint may be relaxed if necessary. Most importantly the algorithm provides a simple means for testing which springs, having negligibly small spring constants, can be omitted without significantly affecting the calculation of the diffuse diffraction patterns.

The final subset of atom–atom interactions with 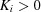 comprise the *effective* intermolecular interactions that are used in the simulations. The use of such effective interactions is necessary to reduce the computational task to a tractable level. The aim is to arrive at a set of springs that are able to mimic the vibrational properties of the real crystal.

#### 3.1.1. Example, *p*-chloro-*N*-(*p*-chlorobenzylidene)aniline   

**Figure 4:**
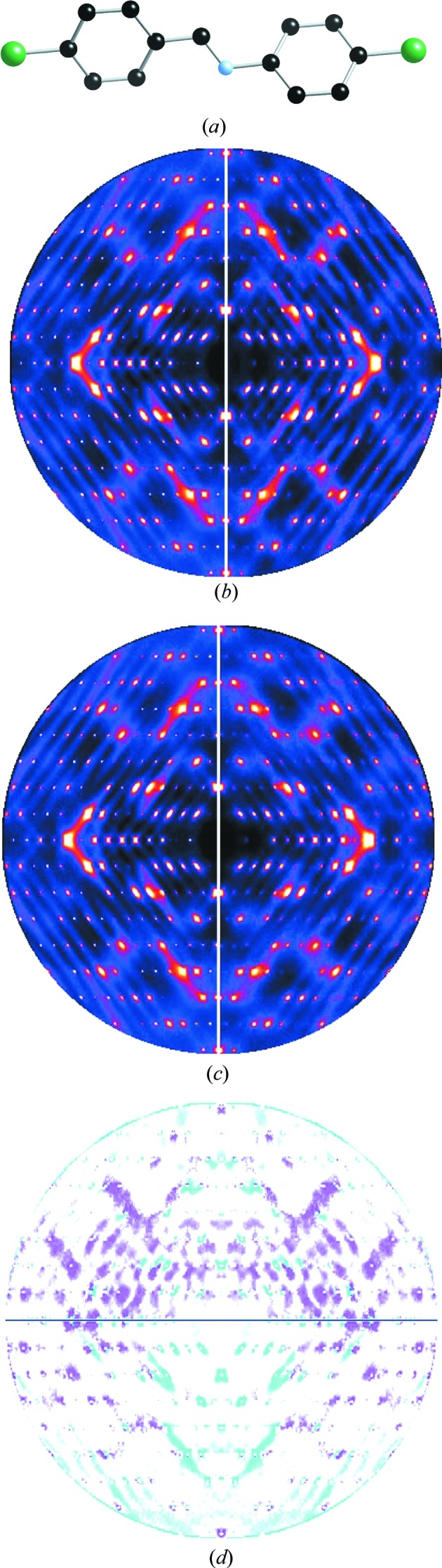
(*a*) The *p*-chloro-*N*-(*p*-chlorobenzylidene)aniline molecule (without H atoms). (*b*) Observed (left) and calculated (right) *hk*0 diffraction patterns for model **I**. (*c*) Observed (left) and calculated (right) *hk*0 diffraction patterns for model **II**. (*d*) Differences 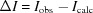 for model **I** (top) and model **II** (bottom).

As an example of the use of this methodology we show in Fig. 4 ▶ some diffraction patterns for two different models of the molecular crystal *p*-chloro-*N*-(*p*-chlorobenzylidene)aniline. The *hk*0 pattern for model **I** is shown in Fig. 4 ▶(*b*) and that for model **II** in Fig. 4 ▶(*c*). Difference plots are shown in Fig. 4 ▶(*d*). In model **I** the molecule is treated as a single rigid body with a geometry derived from the average crystal structure reported by Bernstein & Izak (1976 ▶). For model **II** flexibility was allowed for the dihedral angles in the C—C=N—C bridge.

Calculated diffraction patterns for three sections, *h*0*l*, *hk*0 and 0*kl* of the two models were compared to the observed data. The agreement factors for these sections are given in Table 1 ▶. Although for the *hk*0 section shown in Fig. 4 ▶ the agreement is only slightly better for model **II**, overall this second model with the flexible C—C=N—C bridge is substantially better than model **I**. The plots of the difference between observed and calculated intensities shown in Fig. 4 ▶(*d*) clearly show that despite the overall agreement being very good there is still significant residual intensity, suggesting that the model could be further improved.

The methodology encapsulated in equation (1) and used in the above example has been encoded in the software suite of programs *ZMC* (Goossens *et al.*, 2011 ▶). The program uses a CIF file derived from the average crystal structure determination as a starting point and tools are provided to allow a Monte Carlo simulation model to be set-up in a short time. *ZMC* was designed to allow (relatively) straightforward implementation of a model of a molecular crystal. It allows construction of a model crystal showing displacive, chemical and conformational disorder. Previously, bespoke code had to be written to tackle each new problem, and so *ZMC* has been a useful step on the path to making the study of diffuse scattering a more routine and widespread technique.

#### 3.1.2. Occupancy disorder and size–effect relaxation   

Although the example shown in Fig. 4 ▶ was one showing purely thermal diffuse scattering (TDS), ZMC also includes the option of modelling occupational disorder in which each molecular site is occupied by one or other of two different entities (molecules or molecular orientations). These are specified in terms of a set of spatially interacting binary random variables, 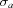. Short-range order can be introduced into this array using a separate Monte Carlo simulation using, as one possibility, an Ising formulation
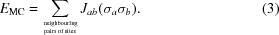



The subsequent introduction of thermal displacements then requires a modification of equation (1) in which the values of the equilibrium distances, 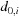, depend on the occupancy of the two connected sites
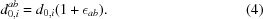
Here 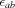 are size–effect parameters whose value depends on the values of 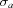 and 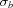.

#### 3.1.3. Summary   

Monte Carlo simulation of molecular crystals has now reached a level of accuracy where quantitative agreement with observed diffuse scattering data may be achieved almost routinely. To achieve this a MC simulation is carried out using a simulation cell of sufficient size (typically 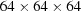 unit cells) that calculated diffraction patterns of a comparable quality to observed patterns may be obtained. In addition the number of MC simulation cycles must be sufficiently large that the final distributions closely approach equilibrium (typically 5000 MC cycles). The use of an empirical formula [equation (2)] to define the spring constants of the harmonic interatomic interactions used in the MC simulation allows a model to be established with only a few adjustable parameters to describe the thermal vibrations. This then allows the further modelling of occupational disorder, size–effect relaxation and any other effects not accounted for by the TDS model.

Even quite subtle effects can be detected. For example, in the comparative study of the two known polymorphs of benzocaine (Chan *et al.*, 2009*b* ▶) it was found that while for Polymorph **I** the scattering could be accounted for by a purely thermal model, for Polymorph **II** there were some residual features observed that proved to be precursor effects of an impending phase transition to a new Polymorph **III** (Chan, Rae & Welberry, 2009 ▶; Chan & Welberry, 2010 ▶). Similarly in a study of the polymorphs of aspirin, the scattering in Polymorph **I** is well described by a purely thermal model but the scattering effects in Polymorph **II** require additional modelling using occupational defects and relaxation around them (Chan, Welberry, Heerdegen & Goossens, 2010 ▶). In the study of ferroelectric triglycine sulfate (Hudspeth *et al.*, 2013 ▶, 2014 ▶) the ability to quickly and accurately model the displacive scattering allowed the weak signals associated with occupancies (in this case flips of glycine molecules) to be isolated, modelled and explained. Because the diffuse streaks associated with the short-range order occur at integer *hkl*, they overlap with tails from large thermal diffuse scattering on the Bragg positions, and so the ability to model the latter proved crucial.

### 3.2. Monte Carlo simulation of disorder in inorganic systems   

The method described in the previous section, in which a network of harmonic springs is used to provide *effective* intermolecular interactions between atoms placed at sites obtained from the average structure determination, has been been particularly successful for molecular crystals. Representing the intermolecular interactions as a sum over atom–atom pairs has a long history of use for molecular cystals (Kitaigorodskii, 1973 ▶; Williams, 1974 ▶) and the methods have developed to become highly sophisticated and accurate tools that are used with effect in crystal structure prediction (Price, 2014 ▶). Nevertheless, the simple empirical atom–atom potentials provide a good first approximation.

Basically the same methodology can be applied to inorganic materials but there are some key differences that must be considered. First, different kinds of bonding must be considered including covalent bonding, ionic bonding, metallic bonding *etc*. There is also the possibility of electronic effects such as Jahn–Teller distortion (Jahn & Teller, 1937 ▶; Proffen *et al.*, 1999 ▶) being important and the generally greater range of interactions in inorganic materials means there is a need to use Ewald summations (Darden *et al.*, 1999 ▶). In principle *ab initio* quantum chemistry methods such as density functional theory (DFT) (Hanaor *et al.*, 2012 ▶) can be used to obtain accurate structural information, but they are still limited in the numbers of atoms that can be handled and are still unable to model disordered systems large enough to provide accurate spatial correlations that can be compared to diffraction data.

In view of all of the above considerations it cannot be expected that simple spring models would be capable of providing quantitative modelling of the diffuse scattering that occurs in these systems. Nevertheless, such models have proved useful in providing qualitative (even semi-quantitative) descriptions of the disorder and associated diffuse scattering for a wide range of materials and so provided insights into the underlying mechanisms. Of particular note have been the elucidation of the complex distribution of defects in the non-stoichiometry oxide wüstite, Fe_1−*x*_O (Welberry & Christy, 1997 ▶), cubic stabilized zirconia (Welberry *et al.*, 1993 ▶) and the aluminosilicate ceramic mullite (Butler & Welberry, 1994 ▶).

In this subsection we give a brief account of studies carried out to elucidate the nature of the disorder in the relaxor ferroelectrics Pb(Zn_1/3_Nb_2/3_)O_3_ (PZN) and Pb(Mn_1/3_Nb_2/3_)O_3_ (PMN). Initially a simple MC model was established that qualitatively reproduces the observed diffuse scattering and this is compared to a more recent study in which it was attempted to use a potential derived from density functional theory to calculate the diffuse scattering *ab initio*.

**Figure 5:**
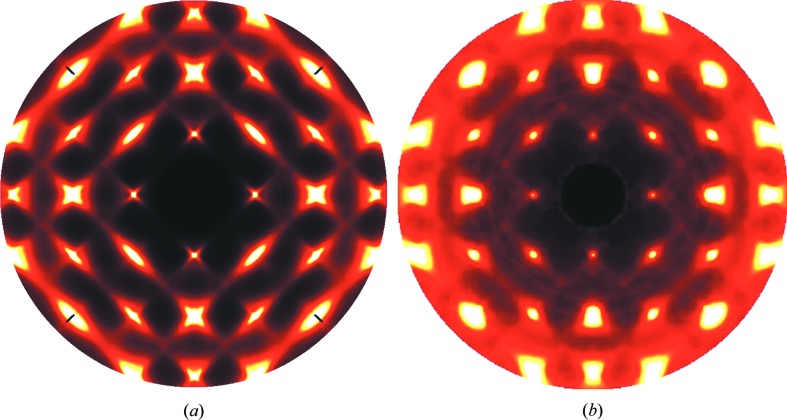
Diffuse scattering in the *hk*0 section of Pb(Zn_1/3_Nb_2/3_)O_3_ (PZN). (*a*) X-ray scattering collected at the 11-ID-B beamline of the Advanced Photon Source (APS). (*b*) Neutron scattering collected on the SXD beamline of the ISIS spallation neutron source.

The diffuse scattering in these (and other similar) relaxors comprises a set of diffuse rods that extend along the rows of Bragg reflections in each of the six 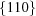 directions. There is a variation of intensity along the rods that is maximum at the Bragg positions resulting in characteristic cross-shaped features where two such sets of rods intersect. Each set of diffuse rods shows an overall distribution of intensity that is *transverse polarized*, meaning that the rod passing through the origin has zero intensity (see Welberry, Gutmann *et al.*, 2005 ▶; Welberry *et al.*, 2006 ▶). Fig. 5 ▶ shows the *hk*0 section for PZN. Fig. 5 ▶(*a*) is X-ray data whereas Fig. 5 ▶(*b*) is neutron data. For X-rays the patterns are dominated by the heavy cations, while for neutrons the oxygen ions play a much greater role.

#### 3.2.1. Simple MC model   

A simple MC model was established on the basis that the driving force for the development of a polar nanodomain structure is the fact that the Pb ions have to displace away from the centre of their coordination polyhedra in order to satisfy their valence requirements. Bond valence calculations (Brese & O’Keeffe, 1991 ▶) indicate that the magnitude of these displacements must be of the order of 0.5 Å. A set of random variables, 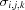, was used to represent the displacements of the Pb ions at each site 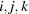 of a simple cubic lattice. 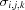 was used to represent a static displacement of ± 0.5 Å: in one or other of the six 〈110〉 directions.

**Figure 6:**
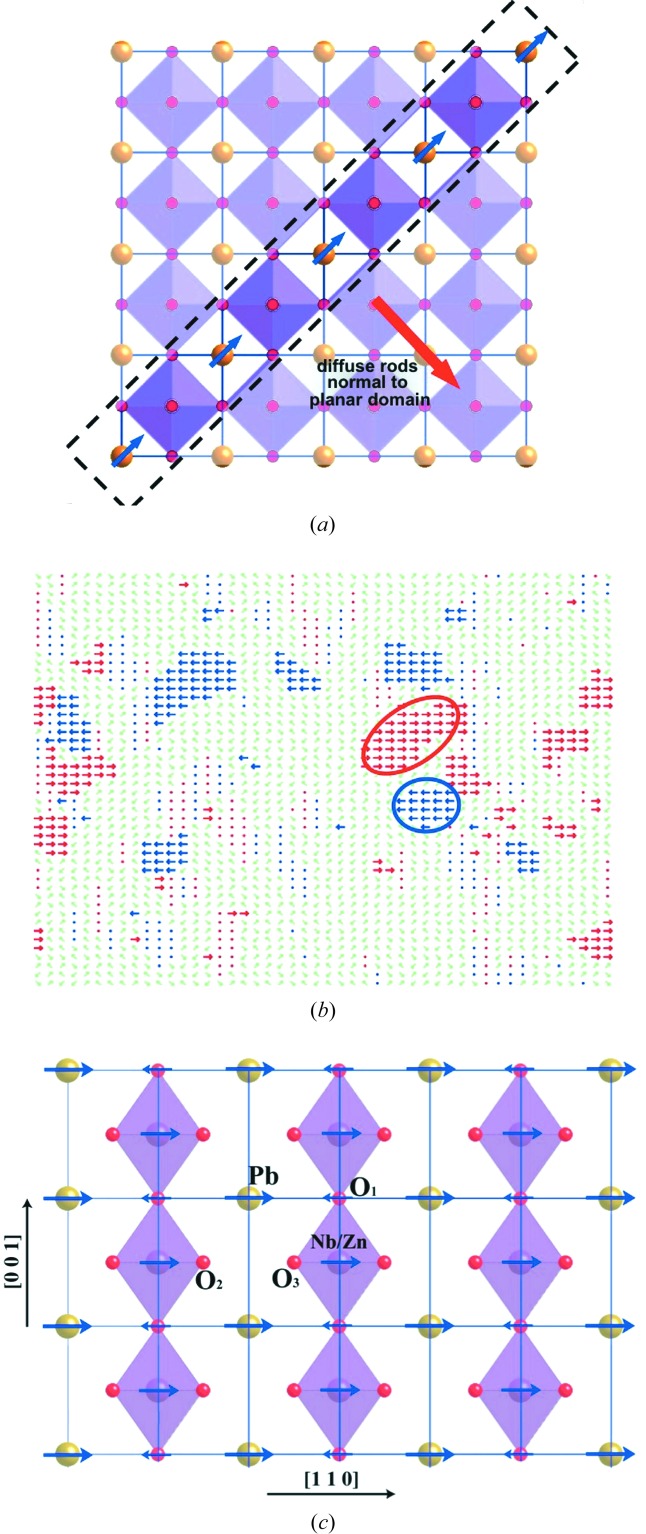
(*a*) A simple model for PZN comprising polar domains oriented normal to each of the six {110} directions. Blue arrows indicate the direction of the displacements of Pb ions from their average position. (*b*) Single layer normal to 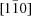 in the resulting simulation array, showing the polar nanoregions (PNRs) that have formed. (*c*) The structure viewed down 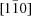 showing the direction of displacement of the different ions in a polar nanodomain (see Welberry, Gutmann *et al.*, 2005 ▶; Welberry *et al.*, 2006 ▶). (*b*) is based on Fig. 4 ▶(*a*) of Welberry *et al.* (2006 ▶).

A second consideration was the knowledge that the Fourier transform of a diffuse rod in reciprocal space must correspond to some kind of planar feature in real space. A simple MC energy was used to promote the development of planar polar nanodomains in which neighbouring Pb displacement vectors within the domains are aligned as shown in Fig. 6 ▶(*a*). By giving no energetic advantage to displacement vectors in adjacent planes, the thickness of the domains is essentially restricted to a single layer. Fig. 6 ▶(*b*) shows a typical distribution of Pb displacements in a single layer normal to 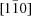 of the resulting simulation array.

Fig. 6 ▶(*a*) shows a schematic view of the Model 1 structure. Planar nanodomains occur normal to each of the six {110} directions. The predominant displacement of the Pb ions lies within the plane and is also directed along a 〈110〉 direction. This pattern of displacements gives rise directly to the *transverse polarized* nature of the diffuse scattering. Consideration of the relative intensity of different orders of the sets of {110} diffuse rods leads to the conclusion that Nb/Zn cation displacements are positively correlated with the primary Pb displacements, whereas the displacement of the oxygen ions are negatively correlated. This is shown in Fig. 6 ▶(*c*).

#### 3.2.2. Further development of simple MC models   

The very simple model described in §3.2.1 reproduces qualitatively all of the observed diffraction features. However, it assumed from the outset that Pb displacements were along 〈110〉 directions and were fixed in magnitude. Subsequent studies (Paściak *et al.*, 2007 ▶; Welberry & Goossens, 2008 ▶) have shown that very similar results can be obtained with models that have 〈100〉-, 〈110〉- or 〈111〉-type displacements. In fact, bond-valence calculations show that Pb can achieve a valence of 2 by displacing by about 0.5 Å, in any direction. All that is necessary to produce 〈110〉-oriented *transverse polarized* diffuse rods is the relative displacement of ions either side of a domain boundary must be parallel to 〈110〉, *i.e.* 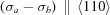.

#### 3.2.3. Atomistic shell model based on *ab initio* calculations   

Although the above approaches lead to models that give DS patterns that agree moderately well with observations, the results are not entirely satisfactory, as the interactions are based on intuitive understanding of the system and not on rigorous *ab initio* calculations. Moreover, this work has also shown that a range of different models can lead to very similar results. In this subsection we describe an attempt to build a fully atomistic model using a potential based on density functional theory calculations (Paściak *et al.*, 2012 ▶). This work was carried out for PMN rather than PZN, but the diffraction patterns for these two sister compounds are very similar.

**Figure 7:**
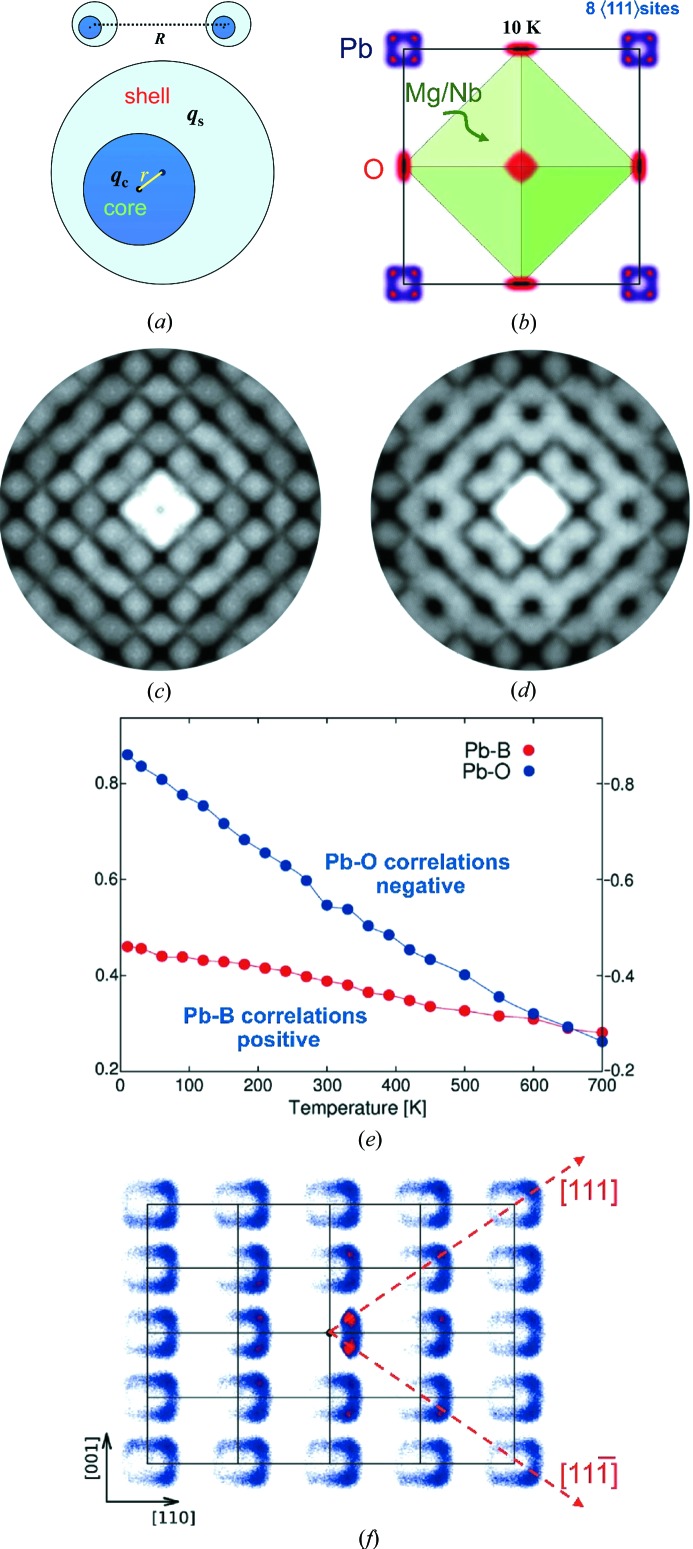
(*a*) Core-shell model definitions. (*b*) Plot of the average unit cell distribution resulting from the MD simulation at 10 K. (*c*) Calculated *hk*0 X-ray pattern. (*d*) Calculated *hk*0 neutron pattern. (*e*) Plots of correlations between the displacements of neighbouring atoms for Pb—(Nb/Mg) and Pb—O. (*f*) Probability plot of the displacement of Pb in the unit cells surrounding a given target cell in which the Pb ion is displaced along 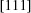 or 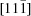. (*b*), (*e*) and (*f*) are based on Figs. 2(*a*), 4(*b*) and 6(*b*) of Paściak *et al.* (2012 ▶).

Molecular dynamics (MD) simulations were carried out using a pair potential based on that developed specifically for PMN by Sepliarsky *et al.* (2005 ▶) and Sepliarsky & Cohen (2011 ▶). Within the model each atom is represented by a core and a shell. There are Coulomb interactions between all cores and shells, except for those within the same atom. For these an anharmonic spring of the form
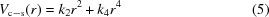
is used, where *r* is a distance between the core and shell (see Fig. 7 ▶ *a*). Short-range interactions are represented by a Rydberg form
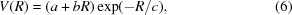
where *R* is the distance between two atoms.

This potential therefore requires specification of constants 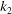 and 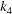 in equation (4), *a*, *b* and *c* in equation (5) as well as the electric charges 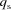 and 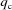 for each atom. The values of these constants were determined from the DFT results, but were further optimized by fitting to Born effective charges derived from the detailed calculations and also by fitting to a number of physical properties including experimental lattice parameters, atomic displacement parameters, bulk and shear moduli, elastic constants and the high-frequency dielectric constant (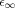). See Paściak *et al.* (2012 ▶) for further details.

Fig. 7 ▶(*b*) shows the distribution of atom positions in the 10 K MD simulation plotted for the average unit cell. It is seen that at low temperatures the Pb site shows a splitting into eight distinct sites each displaced from the average position along one of the 〈111〉 directions. At higher temperatures (300 K) the site distribution maintains a similar overall shape, but now the eight individual sites merge into a single cuboidal shaped distribution. The O ions also have a highly anisotropic shape. Fig. 7 ▶(*c*) shows plots of the way in which the displacements of pairs of neighbouring ions are correlated in the 10 K simulation. These correlations are strongest at low temperature and fall almost linearly with temperature. Notice that while the *B*-site ion displaces in phase with the Pb the displacements of Pb and O are negatively correlated. This correlation behaviour is essentially the same as the pattern of displacements shown in Fig. 6 ▶ for the simple MC model. These correlations give rise to the diffuse scattering and Fig. 7 ▶(*d*) shows a neutron diffraction pattern calculated for the *hk*0 section at 300 K. The form of the pattern at 10 K is similar but rather stronger. The well known features of relaxor SCDS patterns are well reproduced. This is quite remarkable given the potential fitting procedure did not take into account any quantity directly related to DS.

Finally Fig. 7 ▶(*e*) shows a conditional probability plot of the unit cells surrounding a given target unit cell in which the Pb ion is displaced along 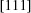 or 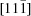. The plot is for a single layer of the structure normal to 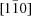. This clearly shows that when the central atom has a component of displacement along 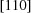 (*i.e.* 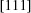 or 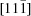) the neighbouring Pb ions both horizontally and vertically have a strong tendency to be displaced in concert with the central Pb. Similar plots of the next layer below show a much reduced tendency for this cooperative behaviour, indicating that the effect occurs in essentially planar domains as envisaged in the simple model of §3.2.1. See Paściak *et al.* (2012 ▶) for further details.

#### 3.2.4. Summary   

The example described in this subsection serves to show that for complex inorganic materials the possibility of calculating the properties (including the diffraction patterns) *ab initio* is still some way off. However, the study described in §3.2.3 is a good first attempt at obtaining such results. It has yielded a fully atomistic model described by an *ab initio*-based potential that realistically reproduces observations made on the real material including the diffraction properties as a function of temperature. Nevertheless, the very simple MC model described in §3.2.1 was a useful stepping stone from which a great deal of insight was obtained and the usefulness of such simple models cannot be overemphasized.

### 3.3. Three-dimensional pair distribution function (3D-PDF)   

In the MC modelling discussed above, a real space model is developed and (sections of) its diffraction pattern calculated and compared to the observations. When data are limited in extent, this is the most likely approach. But now that it is possible to collect large spherical volumes of data, sampling virtually all of the reciprocal space out to some maximum 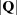, the direct processing of the diffraction data to give details of the real-space atomic distribution is possible. In particular, an approach analogous to pair distribution function analysis of powder diffraction data can be developed.

The powder pair distribution function (PDF) of a sample gives the probability of finding two scatterers at a given distance. Experimentally, it can be obtained from the total scattering (TS) from a sample. If the TS is described by the properly normalized and corrected function 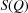, where 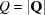 is the scattering vector *magnitude* then
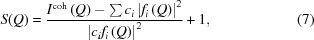
where 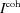 is the observed intensity at *Q*, 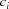 are the atomic concentrations and 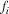 are the X-ray form factors. The pair distribution function, 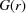, is then calculated from what is effectively a Fourier transform
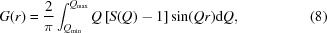
where a choice of 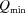 and 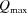 has an influence on the calculated 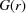  (Proffen & Billinge, 1999 ▶). Note that 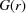 is a function of the pair separation *magnitude*.

The PDF is then a real-space picture of the interatomic separations present in a sample, and does not depend on the material being crystalline or long-range ordered, and hence has found uses in a wide range of studies including nanomaterials and amorphous materials where single crystals are effectively unobtainable.

For systems which are anisotropic, like those which are substantially crystalline, the powder technique inevitably results in loss of information (Goossens & Whitfield, 2014 ▶) due to overlap of features. If instead of a powder pattern a complete TS *single-crystal* data set is Fourier transformed, the 3D-PDF can be obtained; it is rather like calculating a Patterson function except the intensities from *all* reciprocal positions are used, not just integer *hkl*  (Schaub *et al.*, 2007 ▶). Given that the average structure of a crystalline material will tend to dominate the signal, it has been shown that it is often more useful to examine the component of the 3D-PDF calculated from the diffuse intensity only  (Weber & Simonov, 2012 ▶; Schaub *et al.*, 2011 ▶), sometimes referred to as 3D-ΔPDF, although this requires good modelling of the instrument resolution for separation of components, and even then diffuse scattering can be sharp as a Bragg peak in one direction and broad in another  (Weber & Steurer, 2009 ▶).

**Figure 8:**
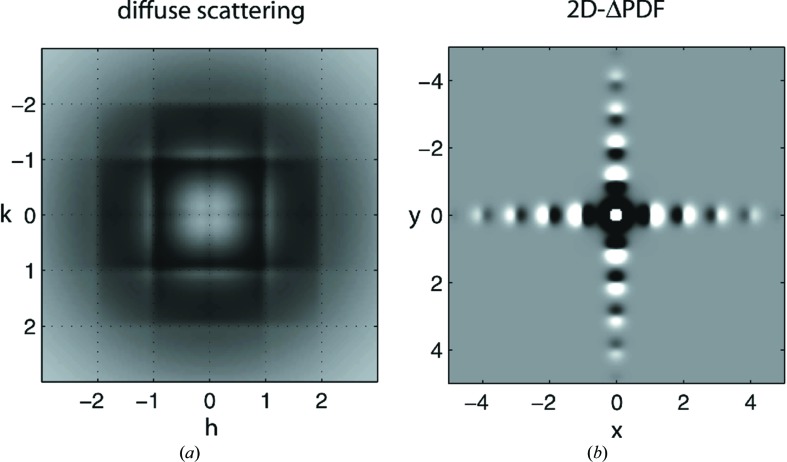
Diffuse scattering and 2D-ΔPDF patterns of the size–effect disorder model. Bragg reflections are not shown, but would be visible at integer *h*; *k* positions according to the *p*-lattice (*a* = 2.5Å). The dotted grid in the diffuse scattering pattern is at integer *h* and *k* values to demonstrate the asymmetry of the diffuse intensities with respect to the integer Bragg positions. Reproduced from Weber & Simonov (2012 ▶) with permission.

The 3D-ΔPDF directly shows variations away from the average, allowing qualitative conclusions about the nature of the disorder to be obtained by inspection of the plots. For example, the 2D-ΔPDF shown in Fig. 8 ▶ plots both SCDS and ΔPDF for a simple numerical model in which size–effect (Welberry, 1986 ▶) atomic shifts occur. While the signature in (*a*) is clear to an experienced worker in diffuse scattering, the probabilities plotted in (*b*) need little interpretation to show that some spacings are now much preferred to others.

Some quantitative insights, such as correlation lengths, can also be obtained (Weber & Simonov, 2012 ▶). This immediacy of interpretation is a very useful attribute for broadening the application of the analysis of diffuse scattering because, despite the power of MC simulation noted above, a major barrier to entry is the determination of the basic nature of the disorder. While MC can model any type of local correlation, the experimenter must choose a model to implement. As a guide to this, and a bridge to more detailed modelling approaches, 3D-PDF may prove invaluable. Different forms of disorder have their ‘fingerprints’ in the 3D-ΔPDF, allowing the PDF to act as a (relatively) straightforward diagnostic tool. Further, the approach has benefits in refining a disorder model. By comparing the PDF of the data with a PDF of a model, the need to repeatedly Fourier transform the model is removed. Whether this is a speed benefit will depend on the details of the modelling and the calculation  (Gutmann, 2010 ▶), but it is now an option.

### 3.4. Refinement strategies: least squares, evolutionary algorithms and more   

It is far from trivial to deduce the nature of the disorder in a crystal. If this can be done, a model can be constructed, possibly using MC. If the model is large enough and realistic enough to provide a good representation of a real crystal, yet does not have too many free parameters, it can be refined against the observed data – usually the observed diffraction but perhaps also the derived 3D-(Δ)PDF.

Hence, the problem becomes one of optimization, with the caveat that as the model is optimized it will periodically need to be redesigned to incorporate new sources of disorder, or model the already-posited forms of disorder differently *etc*.

Such optimization will generally involve the minimization of a 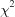 statistic, or of a crystallographic *R*-factor of some kind, as used in Table 1 ▶, by varying the parameters of the model. These could be the parameters in equation (3), or interatomic force constants themselves  (Welberry *et al.*, 1998 ▶, 2001 ▶) or other quantities. In reverse MC, the *R*-factor itself becomes the MC ‘energy’ function, and is minimized within the MC calculation  (Nield *et al.*, 1995 ▶) by directly varying atomic coordinates. Since there are many thousands of atoms in a simulation, this approach means refining a vast number of ‘free’ parameters, and must be approached with caution and an eye to possible constraints that can be applied, but can deliver important insights.

In the case of a ‘forward’ MC model (or similar), the process in broad terms may be outlined as:

(1) Establish the model and its ‘current best’ parameters.
(2) Calculate the SCDS of the model, and then the goodness-of-fit, *R*.
(3) Vary the parameters; usually this means spawning a range of new parameter sets from the current best guess.
(4) Recalculate the SCDS and *R* for each new parameter set.
(5) Use this calculation as a guide for establishing a new set of best parameters
(6) Go to 2.

To this could be added an additional step; if the model cannot be refined to a good enough fit, redesign the model and go to step 1[Other l2li1].

One approach is to use least squares. Allow that the model has *N* parameters that can be written in a vector, 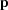. 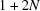 simulations are then performed; in simulation zero *R* for the current model is obtained. In parallel (if enough processors are available!) *N* pairs of simulations are performed. In one simulation within pair *i*, parameter 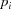 is incremented by 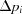, and in the other it is decremented. All other parameters remain fixed. This gives an estimate for 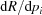. This allows construction of a LS matrix and a new set of ‘best’ parameters can be derived. This is a relatively computationally intensive process that does not work well unless the model is already close to the global minimum, and care must be taken when setting up the diffuse scattering calculation, processing and correcting the observed data, and setting the 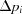. It has been used with some success, both in directly refining force constants  (Welberry *et al.*, 2001 ▶; Goossens *et al.*, 2007*a* ▶,*b* ▶) and in refining the parameters that determine them  (Chan & Goossens, 2012 ▶).

With the limitations of LS in mind, other approaches have been tried. These include various evolutionary algorithms  (Weber & Bürgi, 2002 ▶; Weber, 2005 ▶; Weber *et al.*, 2008 ▶; Bürgi *et al.*, 2005 ▶) and a process called PSO or particle swarm optimization  (Michels-Clark *et al.*, 2013 ▶). All are deigned to sample the parameter space efficiently, avoid local minima and move toward the global minimum.

These more complex approaches have been successful; indeed, it has been concluded that ‘the combination of evolutionary algorithms with Monte Carlo modelling is capable of carrying out structure solution as well as refinement of a disorder model’  (Weber *et al.*, 2008 ▶). This work also compared ‘Differential Evolution’ (DE, outlined in broad terms in Storn & Price, 1997 ▶, and in the context of diffuse scattering in Bürgi & Weber, 2002 ▶) with ‘Cooperative Evolution’ (CE)  (Weber, 2005 ▶), to conclude that DE is the method of choice for refinement, while CE’s ability to avoid ‘getting stuck in a local minimum’ suggests it might be preferable for structure solution, though it is less parallelizable.

Genetic and evolutionary algorithms use an analogy with Darwinian evolution – a generation of ‘individuals’ is spawned from an initial model or set of models, their fitness is tested (in the case of crystallography, usually using some kind of *R*-factor again) and then a new generation is derived from some fraction of the fittest members of the previous according to some rules that mix the parameters, 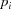 (‘genes’), belonging to each model (this mixing can be done in a number of ways), add in some randomness, and perhaps trim some parameter values according to various criteria.

These rules can be tuned to optimize the algorithm such that it is able to find the global minimum within reasonable time.

By contrast, in PSO  (Kennedy & Eberhart, 1995 ▶) the analogy is with a flock of birds. At each step each ‘bird’ (or particle, or ant) moves through parameter space from one position (effectively, one 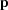) to another. At each step, each individual uses both its own experience of the best 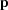, combined with some input from the experiences of the rest of the individuals, to choose its next position. The net result is that the whole flock should gravitate towards the best fit. Some recent results suggest that PSO is useful in the initial phase of the refinement, when wide regions of parameter space need to be explored, but once the models have ‘clustered’ together, DE may be preferable (Michels-Clark *et al.*, 2013 ▶).

In all these approaches, a central issue is how the previous generation of solutions is used to derive the next.

The net result is that the best approach may be a combination of methods; CE and PSO seem to have strengths in finding the region of the global minimum in *R*, while DE offers a faster means of refining a good solution, as does LS when close to the global minimum. The problem remains of deciding what aspects of the structure need to be parameterized at all (in other words, what is the nature of the local order or what is happening in the structure that needs to be modelled?). Here 3D-PDF, combined with interactive, qualitative modelling of aspects of disorder, is the most likely route.

Up until now, the parameters in equation (3) have been optimized using LS, but they could just as well be optimized using the other methods mentioned here. In all cases, the core of the problem is a robust, reliable way of defining and calculating the *R*-factor. As in crystallography more widely, weighting schemes, criteria for including or omitting data (for example, how to decide whether a pixel belongs to the Bragg peak), the ability to model instrumental resolution and assumptions regarding the form of the background will all affect the calculation of *R* and therefore the result of the optimization process.

## 4. Conclusion   

Today, radiation sources, detectors and data reduction software allow the collection of high-quality, three-dimensional SCDS patterns. Short-range order of virtually any nature or degree of complexity can be modelled using a Monte Carlo model. For the user new to diffuse scattering, difficulty arises in determining what aspects of the structure are disorded and in what way, *i.e.* what should the MC model ‘look like’? While it is not yet possible to use highly exact calculations (such as DFT) to model a system large enough to demonstrate the nanoscale correlations apparent in SCDS, such calculations can now be used to guide modelling approaches (MC, RMC *etc.*) that *can* be used on large systems. Calculation of the 3D-PDF can similarly help. This is valuable in expanding the reach of the technique. Conventional diffraction, and now PDF, have become techniques that scientists from Physics to Medicine can draw on to inform their studies. As computers grow in power and new analysis approaches are developed, less specialized researchers will be able to make use of SCDS, allowing it to have wider impact.

## Figures and Tables

**Table 1:** Agreement factors, 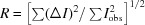, for individual sections for the fitting of models **I** and **II** The *hk0* sections are shown in Fig.4 ▶.

Section	Model **I**	Model **II**	No. of pixels
*hk*0	0.249	0.232	115092
0*kl*	0.240	0.180	118632
*h*0*l*	0.358	0.260	101852
